# CryoTransformer: a transformer model for picking protein particles from cryo-EM micrographs

**DOI:** 10.1093/bioinformatics/btae109

**Published:** 2024-02-24

**Authors:** Ashwin Dhakal, Rajan Gyawali, Liguo Wang, Jianlin Cheng

**Affiliations:** Department of Electrical Engineering and Computer Science, University of Missouri, Columbia, MO 65211, United States; NextGen Precision Health, University of Missouri, Columbia, MO 65211, United States; Department of Electrical Engineering and Computer Science, University of Missouri, Columbia, MO 65211, United States; NextGen Precision Health, University of Missouri, Columbia, MO 65211, United States; Laboratory for BioMolecular Structure (LBMS), Brookhaven National Laboratory, Upton, NY 11973, United States; Department of Electrical Engineering and Computer Science, University of Missouri, Columbia, MO 65211, United States; NextGen Precision Health, University of Missouri, Columbia, MO 65211, United States

## Abstract

**Motivation:**

Cryo-electron microscopy (cryo-EM) is a powerful technique for determining the structures of large protein complexes. Picking single protein particles from cryo-EM micrographs (images) is a crucial step in reconstructing protein structures from them. However, the widely used template-based particle picking process requires some manual particle picking and is labor-intensive and time-consuming. Though machine learning and artificial intelligence (AI) can potentially automate particle picking, the current AI methods pick particles with low precision or low recall. The erroneously picked particles can severely reduce the quality of reconstructed protein structures, especially for the micrographs with low signal-to-noise ratio.

**Results:**

To address these shortcomings, we devised CryoTransformer based on transformers, residual networks, and image processing techniques to accurately pick protein particles from cryo-EM micrographs. CryoTransformer was trained and tested on the largest labeled cryo-EM protein particle dataset—CryoPPP. It outperforms the current state-of-the-art machine learning methods of particle picking in terms of the resolution of 3D density maps reconstructed from the picked particles as well as F1-score, and is poised to facilitate the automation of the cryo-EM protein particle picking.

**Availability and implementation:**

The source code and data for CryoTransformer are openly available at: https://github.com/jianlin-cheng/CryoTransformer.

## 1 Introduction

Cryo-electron microscopy (cryo-EM) is a modern biophysical technique used to reconstruct 3D structures from 2D images of biological macromolecules, such as proteins and viruses at cryogenic temperature (Glaeser 2013, Gyawali *et al.* 2023). These 2D images are stored in various formats (like mrc, tiff, tbz, eer, etc.), which are also called micrographs. Given the inherent challenges of ascertaining the orientations of the particles and the low signal-to-noise ratio (SNR) of micrographs, hundreds of thousands of protein particles are often required to determine a high-resolution 3D structure of the protein. These 3D structures of proteins are important for understanding their biological functions and their interactions with ligands (Dhakal *et al.* 2022, Giri and Cheng 2023) facilitate structure-based drug discovery (Dhakal *et al.* 2022, Dhakal *et al.* 2023c). Since the SNR of micrographs is generally low, thousands of micrographs need to be collected to obtain a high-resolution structure for a protein, from which as many as millions of protein particles are picked. Precise identification of true particles is important, as the presence of false positive particles complicates the down-stream 3D protein reconstruction process. The particle picking task is inherently challenging due to several factors, including high noise levels caused by ice and contamination, low contrast of particle images, heterogenous conformations of particles, and variation in the orientation of particles.

This manual particle picking process by human is laborious, tedious, and time-consuming, which cannot be applied to pick millions of particles from thousands of micrographs. Therefore, substantial efforts have been put to develop semi-automated or fully automated methods to pick protein particles, which can be classified into two categories: (i) template-based particle picking and (ii) machine learning particle picking. In template-based particle picking, particle identification relies on comparing potential particles to predefined reference templates. However, due to noise in micrographs this method is often unable to detect particles of unusual shape and suffers from high false-positive rates. Machine learning particle picking consists of both unsupervised learning (clustering) methods (Al-Azzawi *et al.* 2019) and supervised methods (Mallick *et al.* 2004, Langlois *et al.* 2014, Heimowitz *et al.* 2018, Al-Azzawi *et al.* 2020). Recent advancements in deep learning, including XMIPP (Marabini *et al.* 1996), DeepPicker (Wang *et al.* 2016), DeepEM (Zhu *et al.* 2017), Xiao *et al.*’s method (Xiao and Yang 2017), Warp (Tegunov and Cramer 2019), HydraPicker (Masoumzadeh and Brubaker 2020), McSweeney *et al.*’s method (McSweeney *et al.* 2020), DRPnet (Nguyen *et al.* 2021), CrYOLO (Wagner *et al.* 2019), and Topaz (Bepler *et al.* 2019) have automated protein particle picking. Among them, CrYOLO and Topaz, based on convolutional neural networks, are widely used. However, they have been trained with limited particle data. CrYOLO and Topaz were trained on 840 and 2296 micrographs respectively, which is 83.7% and 55.6% less than 5172 micrographs employed to train CryoTransformer in this work. The limited amount of training data makes it difficult for them to generalize to new protein types or shapes.

Topaz and CrYOLO both are based on traditional CNN architectures. Specifically, Topaz follows a positive unlabeled (PU) Learning approach of training with a limited number of sparsely labeled particles and an absence of labeled negatives, while CrYOLO employs the YOLO architecture for the identification of protein particles in cryo-EM micrographs. Topaz uses a sliding window classification of micrographs and extracts the protein particles by using non-maximum suppression technique. CrYOLO applies 22 convolutional and 5 max-pooling layers for feature extraction. It includes a passthrough layer positioned between the 13th and 21st layer to leverage fine-grain features. Following this, the network is concluded with a 1 × 1 convolutional layer for particle detection. For instance, CrYOLO usually overlooks many true protein particles, while Topaz often picks false positives such as ice contaminants and false particles in carbon areas.

To overcome these obstacles, we devised a transformer-based particle picking approach and trained it on the largest, diverse, manually labeled CryoPPP protein particle dataset (Dhakal *et al.* 2023a, b). Inspired by Meta’s Detection Transformer (DETR) (Carion *et al.* 2020) for detecting small objects, we designed the end-to-end detection transformer named as CryoTransformer. Briefly, it has an initial step of reducing noise in micrographs, followed by the feature extraction through a ResNet-152 architecture. Subsequently, a transformer model is used for detecting protein particles. This is succeeded by the feed-forward networks to predict particles, which are followed by the post-processing procedures. Refer to Supplementary Fig. S1 for the overview of the pipeline. The output includes particle markings on the micrographs and the particles coordinates in *.star* files, which can be directly used for the subsequent stages of 3D protein structure reconstruction. We conducted a rigorous evaluation of CryoTransformer and it outperforms the two popular deep learning methods: CrYOLO and Topaz. The source code and data for CryoTransformer are openly available at: https://github.com/jianlin-cheng/CryoTransformer.

## 2 Materials and methods

### 2.1 Dataset

#### 2.1.1 Dataset acquisition

We utilized the largest comprehensive CryoPPP dataset (Dhakal *et al.* 2023a, b) curated from Electron Microscopy Public Image Archive (EMPIAR) (Iudin *et al.* 2023), to train, validate, and test CryoTransformer. The micrographs of 22 proteins (EMPIAR IDs) from the CryoPPP dataset were used, with the data of each EMPIAR ID split according to an 80%-10%-10% ratio for training, validation, and internal test. Moreover, we used the data of six distinct EMPIAR IDs in CryoPPP dataset different from the 22 proteins above as well as the four complete micrograph datasets from EMPIAR repository (Iudin *et al.* 2023) as the independent test dataset to compare CryoTransformer with the external methods.

The selection of training and test data considered a range of protein attributes, including type, shape, size, and overall structural characteristics. The statistics and information of 22 proteins used for the training, validation and internal test are described in Supplementary Table S1. Moreover, Supplementary Fig. S2 illustrates the varying defocus values of the training data. The datasets encompass various protein categories, such as transport proteins, membrane proteins, viral proteins, ribosomes, signaling proteins, aldolases, and more. They comprised micrographs featuring diverse attributes, including those with ice patches, contaminants, varying ice thickness, and carbon areas. Different protein distribution patterns, including monodisperse, clumped clusters, and heterogeneous views, are also included. The Supplementary Tables S2 and S3 contain the information and statistics of the proteins in the independent test dataset.

#### 2.1.2 Denoising and pre-processing of cryo-EM micrographs

In the CryoTransformer image processing pipeline, cryo-EM micrographs in .mrc format undergo several key steps for noise reduction and enhancement. Initially, a Gaussian filter is applied to reduce noise, followed by standard normalization to center and scale pixel values. These normalized images are converted to grayscale for uniform representation. Noise reduction involves a two-step process: Fast Non-Local Means (FastNLMeans) denoising to preserve details and a subsequent Weiner filter to further reduce noise. Contrast Limited Adaptive Histogram Equalization (CLAHE) is used to enhance contrast, addressing non-uniform illumination and low contrast. Finally, guided filtering is performed using the CLAHE-enhanced image as a guide to selectively smooth while preserving fine structural details, resulting in balanced noise reduction and structural information preservation. In depth denoising pipeline is described in Supplementary Fig. S3 and Supplementary Note S1.

#### 2.1.3 Generating COCO-dataset for labeled protein particles in micrographs

We used the ground truth particle coordinate data from the CryoPPP dataset (Dhakal *et al.* 2023a, b) to generate labels to train CryoTransformer. The particle labels were stored in Common Objects in Context (COCO) format (Lin *et al.* 2014). An illustration of how these labels are stored is depicted in Supplementary Fig. S3 (H). In the case of all training and validation images, we have two JSON files: one for training (referred to as the ‘train JSON’) and another for validation (referred to as the ‘validation JSON’). For each particle, we retain details such as its bounding box coordinates, area, category label (typically set to 1 in our case as all objects to be detected are protein particles), the corresponding image reference, and a unique particle ID.

### 2.2 Design and implementation of CryoTransformer

CryoTransformer is designed to achieve the accurate prediction of bounding boxes for the protein particles within a micrograph, while minimizing the number of false positives. It undergoes an end-to-end training, using a specialized loss function that effectively combines the bipartite matching loss between predicted and ground-truth protein particles in the micrographs.

#### 2.2.1 CryoTransformer architecture

**Figure 1.**
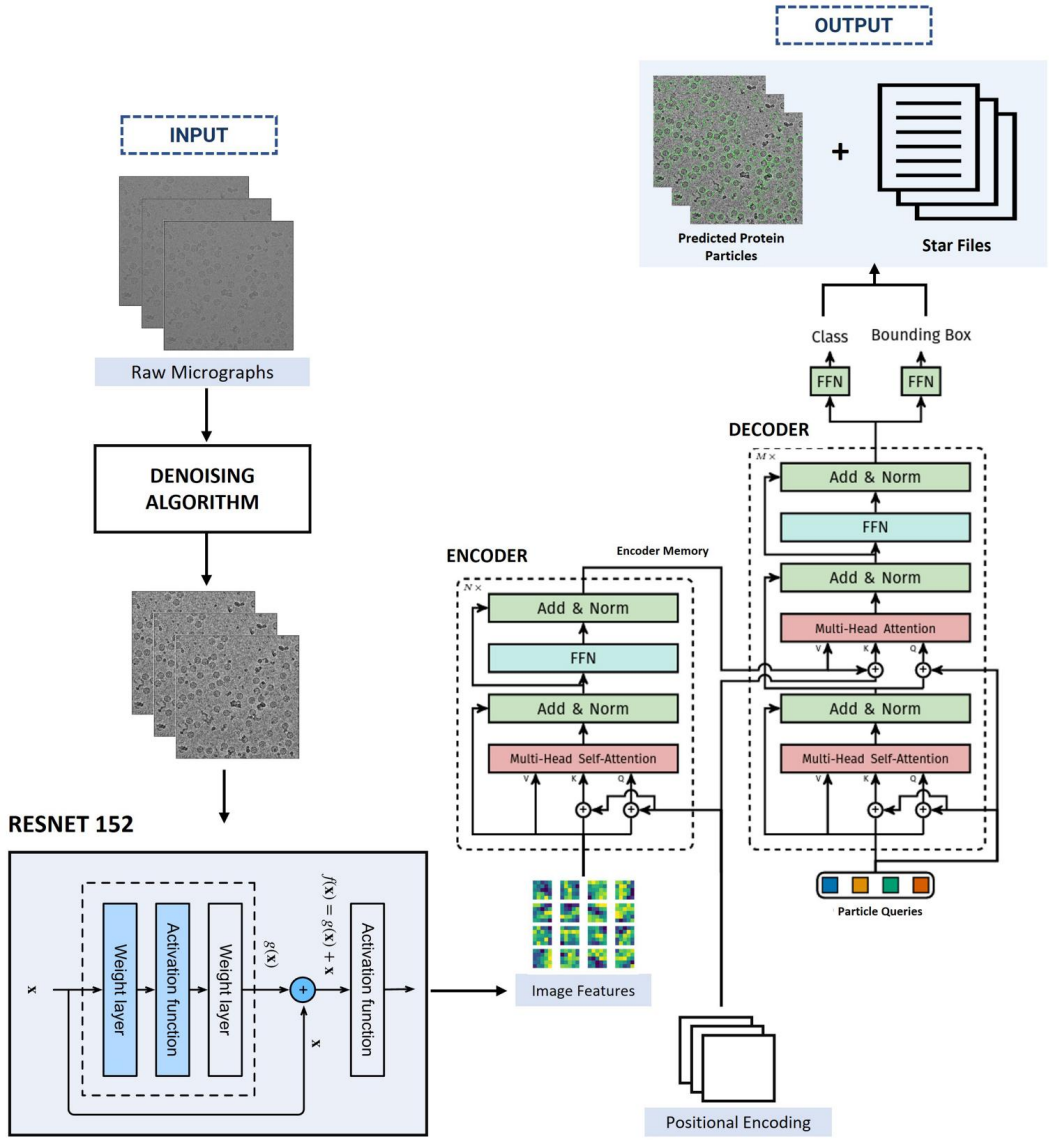
Architecture of CryoTransformer. The raw micrographs are denoised and are fed into the ResNet-152 module for feature extraction. The image features, along with positional encoding, are fed to the encoder of the transformer (Carion *et al.* 2020). The output from the encoder is subsequently passed to the decoder layer. Finally, the decoder’s output is passed to the feed forward networks that generate the protein particle bounding box predictions. The final output includes the visual representation of predicted protein particles encircled in micrographs and .star files.

As illustrated in Fig. 1, CryoTransformer comprises three main components: a Convolutional Neural Network (CNN) with residual connections [Resnet-152 (He *et al.* 2016)] responsible for feature extraction, an encoder-decoder transformer (Vaswani *et al.* 2017, Carion *et al.* 2020) for learning the shapes of the particles in the context of an entire image, and a feed-forward network (FFN) responsible for producing the ultimate particle predictions.

##### 2.2.1.1 Resnet-152 backbone block

The Resnet-152 receives the preprocessed micrographs $x_{\text{img}}\in R^{3\times H_{0}\times W_{0}}$ (with three color channels)) as input and generates a lower-resolution activation map as $f\in R^{C\times H\times W},$ Where C=2048, and $H=\frac{H_{0}}{32}$, $W=\frac{W_{0}}{32}$. 0 padding is applied to the images in a batch to make sure that they all have same input dimensions $\left(H_{0},W_{0}\right)$ as the largest image size of the batch.

##### 2.2.1.2 Transformer module

###### Transformer encoder

The features extracted from the Resnet-152 are subsequently passed through the encoder of the Transformer. The encoder plays a vital role in generating coherent and context-aware outputs. In the encoder, a 1x1 convolution operation is used to decrease the channel dimension of the high-level activation map, denoted as f, from C to a smaller dimension d, yielding a new feature map $z_{0}\in R^{d\times H\times W}$. Since the encoder accepts a 1D sequence as input, we collapse the spatial dimensions of $z_{0}$ into a single dimension. As a result, the resultant input becomes a feature map of dimension d×HW. Here, every encoder layer follows a consistent structure, comprising a multi-head self-attention component and a FFN layer. To account for the permutation-invariant nature of the transformer architecture, we enhance it by incorporating the positional encodings (Parmar *et al.* 2018, Bello *et al.* 2019), which are included in the input of every multi-head self-attention layer.

###### Transformer decoder

The decoder receives the memory from encoder, positional encoding, and particle queries as input. It involves the transformation of N embeddings of size d (in our specific scenario, N = 600, meaning predicting max 600 protein particles per micrograph) through the multi-headed self- attention mechanisms. It’s worth noting that since the decoder is also designed to be permutation-invariant, it requires distinct particle queries (initialized as random vectors) within the set of N inputs to generate different outcomes. These particle queries, added to the input at each attention layer, are a set of learnable embeddings which are updated through back propagation. Subsequently, the output of the decoder is individually used to predict box coordinates and class labels (1 in our case) through a feed-forward network, a process detailed in the following subsection, resulting in N final predictions.

##### 2.2.1.3 Feed-forward networks module

The final prediction is generated through a 3-layer perceptron with a ReLU activation function and d hidden nodes in each hidden layer, followed by a linear projection layer. This FFN is responsible for predicting the normalized center coordinates, height, and width of the bounding box relative to the input micrograph. Additionally, the linear layer predicts the class label using a softmax function. Considering that we are making predictions for a fixed-size set of N potential bounding boxes, and N is typically much larger than the actual number of protein particles in a single micrograph, we introduce a special class label denoted as ∅. This label means that no protein particle has been detected in a particular slot. Its role is akin to the ‘background’ class in conventional object detection.

#### 2.2.2 Loss function

CryoTransformer generates a consistent set of N predictions in a single traversal of the decoder. This number N was deliberately chosen to exceed the usual count of protein particles in a micrograph. To achieve this, the loss function is designed to establish an ideal bipartite matching between the predicted protein particles and their corresponding ground truth. Subsequently, the model optimizes the losses pertaining to individual particles in order to refine the predictions further.

We can represent the ground truth set of particles as y and the set of N predictions as $\hat{y}={\left\{{\hat{y}}_{i}\right\}}_{i=1}^{N}$. When N exceeds the number of true protein particles in the micrograph, we enlarge y as a set of size N, with padding represented by ∅ (no protein particle). To find the optimal bipartite matching (Carion *et al.* 2020) between these two sets, we aim to find a permutation of N elements denoted as $\sigma \in S_{N}$ that incurs the lowest cost. This permutation is determined by the following equation, given in Equation (1):

(1) $$\hat{\sigma }=\underset{\sigma \in S_{N}}{\arg\min}\sum_{i}^{N}\;L_{\text{match}\;}\left(y_{i},{\hat{y}}_{\sigma (i)}\right)$$

$L_{\text{match}\;}\left(y_{i},{\hat{y}}_{\sigma (i)}\right)\;$represents the pairwise matching cost between the ground truth particle $y_{i}$ and a prediction indexed by σ(i). This cost is calculated using the following equation (2):

(2) $$L_{\text{match}\;}\left(y_{i},{\hat{y}}_{\sigma \left(i\right)}\right)=\;-1_{\left\{c_{i}\neq \emptyset \right\}}{\hat{p}}_{\sigma (i)}\left(c_{i}\right)+1_{\left\{c_{i}\neq \emptyset \right\}}L_{\text{box}\;}\left(b_{i},{\hat{b}}_{\sigma (i)}\right)$$

We can view each element i in the ground truth set as a $y_{i}=\left(c_{i},b_{i}\right)$, where $c_{i}$ represents the target class label, and $b_{i}$ belongs to the range $[0,1{]}^{4}$, representing a vector that specifies the center coordinates of the ground truth box, along with its height and width relative to the micrograph dimensions. This approach ensures a one-to-one matching, preventing duplicate predictions when directly predicting sets.

The next stage involves calculating the Hungarian loss using the Hungarian algorithm (Stewart *et al.* 2015) for all pairs that were matched in the preceding step. We define this loss according to the Equation (3):

(3) $$L_{\text{Hungarian}\;}(y,\hat{y})=\sum_{i=1}^{N}\;\left[-\log{\hat{p}}_{\hat{\sigma }(i)}\left(c_{i}\right)+1_{\left\{c_{i}\neq \emptyset \right\}}L_{\text{box}\;}\left(b_{i},{\hat{b}}_{\hat{\sigma }}(i)\right)\right]$$

Here, $\hat{\sigma }$ represents the optimal assignment obtained from the initial equation (1).

In practical implementation, we apply a down-weighting factor of 10 to the log-probability term when $c_{i}$ is equal to ∅, denoting the absence of a particle. This adjustment is made to address the issue of class imbalance. The second part of the Hungarian loss ($L_{\text{box}\;}(\cdot ))$ scores the bounding boxes is given by the Equation (4):

(4) $${L_{\text{box}\;}\left(b_{i},{\hat{b}}_{\sigma (i)}\right)=\;\lambda }_{\text{iou}\;}L_{\text{iou}\;}\left(b_{i},{\hat{b}}_{\sigma (i)}\right)+\lambda_{L1}∥b_{i}-{\hat{b}}_{\sigma (i)}{∥}_{1}$$

Where $\lambda_{\text{iou}\;},\lambda_{L1}\in R$ are hyperparameters and $L_{\text{iou}\;}(\cdot )$ is the generalized IoU (Rezatofighi *et al.* 2019) given by Equation (5):

(5) $$L_{\text{iou}\;}\left(b_{\sigma (i)},{\hat{b}}_{i}\right)=1-\left(\frac{\left|b_{\sigma (i)}\cap {\hat{b}}_{i}\right|}{\left|b_{\sigma (i)}\cup {\hat{b}}_{i}\right|}-\frac{\left|B\left(b_{\sigma (i)},{\hat{b}}_{i}\right)\setminus b_{\sigma (i)}\cup {\hat{b}}_{i}\right|}{\left|B\left(b_{\sigma (i)},{\hat{b}}_{i}\right)\right|}\right)$$

In the context provided, |.| denotes ‘area,’ and we use the terms union and intersection of box coordinates as shorthand references for the boxes themselves. To compute the areas of unions or intersections, we rely on the minimum/maximum of linear functions involving $b_{\sigma (i)}$ and ${\hat{b}}_{i}$. This approach ensures that the loss behaves in a stable manner for the computation of stochastic gradients. $B\left(b_{\sigma (i)},{\hat{b}}_{i}\right)\;$refers to the largest bounding box that contains both $b_{\sigma (i)},{\hat{b}}_{i}$.

#### 2.2.3 Model implementation and training

We trained CryoTransformer with AdamW optimizer (Rezatofighi *et al.* 2019) by setting the initial transformer’s learning rate to 10^−4^, the backbone’s to 10^−5^, and weight decay to 10^−4^. All weights are randomly initialized with Xavier initialization (Glorot and Bengio 2010). Additive dropout of 0.1 is applied after every multi-head attention and FFN before layer normalization. We use a training schedule of 300 epochs with a learning rate drop by a factor of 10 after 200 epochs, where a single epoch is a pass over all training images once. Training the model for 300 epochs on NVIDIA A100 80GB GPU took 2 days and 11 hours to complete.

### 2.3 Ablation studies

We performed ablation studies to analyze the performance of the model with (i) different convolution backbone architectures and (ii) different datasets [denoised versus non-denoised (raw) micrographs]. The model configuration and tunned hyperparameter for both the backbone and transformer components of the model are detailed in Supplementary Table S4.

#### 2.3.1 Ablation study with varying convolutional backbone architectures

We conducted various experiments by altering the backbone architectures (ResNet-18, ResNet-34, ResNet-50, ResNet-101, and ResNet-152) to examine their influence on the model's loss functions. These experiments were carried out for both the non-denoised dataset (see Supplementary Fig. S4) and denoised one (see Supplementary Fig. S5). We found that ResNet-152 demonstrated the highest performance, while ResNet 18 exhibited the least favorable outcomes across all evaluated loss functions in both the cases. Refer to Supplementary Tables S5 and S6 for more details.

#### 2.3.2 Ablation study with denoised versus non-denoised datasets

Using the ResNet-152 backbone architecture, we conducted an ablation study to investigate the impact of denoising micrographs on particle picking tasks. This was performed by using two datasets (Denoised versus Non-Denoised) on the CryoTransformer model. We observed that denoising the micrographs reduces the overall training loss by 40.16% and overall validation loss by 34.56%. The detailed statistical results (Supplementary Fig. S6 and Supplementary Table S7) affirm that denoised data consistently outperforms non-denoised (raw) data across all evaluated loss functions.

### 2.4 Postprocessing predictions and reconstructing protein density maps from picked particles

The FFN module of CryoTransformer predicts the coordinates of particles and their corresponding confidence scores (ranging from 0 to1). These predictions are processed in a few steps to generate *.star* file which undergo various operations to build 3D density maps of proteins. The visual representation of the overall process is shown in Supplementary Fig. S7.

The predictions are first used to generate individual box files for every micrograph for a protein, containing the center coordinates (x and y) of all the predicted protein particles. We retain only the particles whose confidence score falls in the range from 25th percentile to 100th percentile. Subsequently, these box files are merged to create a *.star* file that can be accepted by CryoSPARC (Punjani *et al.* 2017) for density map construction for the protein.

The star files generated are imported into CryoSPARC through the ‘import particles’ task, accompanied by input parameters such as Acceleration Voltage (kV), Spherical Aberration (mm), and Pixel Size (Å) as well as the patch-based Contrast Transfer Function (CTF)-estimated micrographs. Subsequently, these particles are extracted using a specified extraction box size (in pixels) and fed into the 2D classification function of CryoSPARC to group them into different orientation classes.

In Cryo-EM single particle analysis, the ‘Select 2D’ step plays a pivotal role in enhancing the quality of protein particle selection, thereby contributing to the improved results. This step is strategically positioned after the initial particle picking process and before the subsequent 3D reconstruction. The significance of the ‘Select 2D’ step lies in its ability to discern true protein particles from some noise or artifacts, thereby mitigating the impact of potential false positives introduced during particle picking. The selection process involves classifying extracted particle images into 2D classes, leveraging reference-free alignment techniques. By performing this classification, valuable insights into the inherent structural heterogeneity within the dataset can be gained. This meticulous selection process ensures that mostly true protein particles with distinct structural classes are utilized in the final 3D density map reconstruction.

To assess the quality of the particles picked by CryoTransformer, CrYOLO and Topaz, we carried out the density map reconstruction experiments with and without the 2D selection respectively. When the 2D classification was used, we generated a total of 50 particle classes, employing a window inner radius of 0.85 and an outer radius of 0.99. Additionally, we performed 15 iterations to refine the CryoSPARC’s noise model. The selected particles were used by an *ab initio* reconstruction process with the standard parameter settings, which includes 300 iterations of reconstruction with a Fourier radius step of 0.04, a momentum of 0 and an initial learning rate of 0.4 for the stochastic gradient descent optimization. Additionally, a lowpass filter cutoff in Fourier radii of 7 was applied to the initial random structures.

After generating the initial density map for a protein, the cryoSPARC's ‘homogeneous refinement’ job was employed to enhance it further. The homogeneous refinement was applied to correct the higher-order aberrations and to refine particle defocus caused by factors such as beam tilt and spherical aberration. To ensure the fairness in comparisons of the particle picking methods, the experiment was conducted three times for each method with different random seed values, and the best score (in Angstrom units) out of the three experiments was used in the comparison.

## 3 Results

We evaluated the particle picking performance of CryoTransformer in the following complementary ways. First, we compared it with CrYOLO and Topaz in terms of the resolution of the 3D density maps reconstructed from the particles picked by them from the full set of micrographs in the EMPIAR repository for the four proteins in the independent test dataset. Second, we compared it with CrYOLO and Topaz in terms of the resolution of the density maps picked from a subset of labeled micrographs in the CryoPPP dataset for the proteins in the independent test dataset. In all the comparisons, we employed CrYOLO's Generic model (publicly available) and Topaz's default model integrated into CryoSPARC (refer to Supplementary Table S8 for parameter details).

Compared to CrYOLO and Topaz, CryoTransformer is unique in utilizing transformers and a bipartite matching loss for direct set prediction. Its architecture eliminates the need for manually crafted components such as a non-maximum suppression procedure or anchor generation. With an optimized COCO-format dataset, robust automated denoising, and loss functions based on bipartite matching (refer to Equations 1–5), CryoTransformer directly produces the final set of predictions that results in high-quality picked particles.

### 3.1 Comparing CryoTransformer, CrYOLO, and Topaz in terms of resolution of density maps reconstructed from the particles picked from the full set of micrographs in the EMPIAR repository (∼1600 micrographs per protein)

The full set of micrographs in the EMPIAR repository for the four test proteins [Human HCN1 Hyperpolarization-Activated Channel (EMPIAR 10081), Influenza Hemagglutinin (EMPIAR 10532), mechanotransduction channel NOMPC (EMPIAR 10093), and asymmetric αVβ8 (EMPIAR 10345)] in the independent test dataset were used to compare CryoTransformer, CrYOLO and Topaz. The resolution of the density map reconstructed from the particles picked by each method for each protein was calculated. The density maps were reconstructed by CryoSPARC in two modes: with 2D particle selection (*Select 2D*) or without it. The experiment for each method and each protein was conducted three times and the best results were selected for the comparison. The comparative results of the three methods are summarized in Table 1, while the detailed results of each trial are reported in Supplementary Table S9.

**Table 1.** Comparison of CryoTransformer with crYOLO and Topaz’s performance in terms of the resolution of density maps reconstructed from the particles picked from the full set of micrographs of the four test proteins.

EMPIAR ID	Number of micrographs	Without select 2D						With select 2D					
		Number of particles			3D resolution (Å)			Number of particles			3D resolution (Å)		
		CrYOLO	Topaz	CryoTransformer	CrYOLO	Topaz	CryoTransformer	CrYOLO	Topaz	CryoTransformer	CrYOLO	Topaz	CryoTransformer
10081 (Lee and MacKinnon 2017)	997	59 559	383 558	293 980	7.45	6.34	**4.89**	32 472	148 378	147 662	6.39	4.19	**4.15**
10532 (Tan and Rubinstein 2020)	1556	62 732	1 574 179	764 215	8.34	3.97	**3.86**	16 079	260 266	259 757	7.82	3.27	**3.21**
10093 (Jin *et al.* 2017)	1873	53 482	791 064	596 192	6	**4.72**	6.11	40 374	359 619	204 355	5.57	**4.37**	4.65
10345 (Campbell *et al.* 2020)	1644	19 836	396 882	182 397	7.27	**3.5**	5.22	5377	155 023	111 375	6.06	3.47	**3.45**

Bold font denotes the best resolution of the density map reconstructed from picked particles in the three trials.

**Figure 2.**
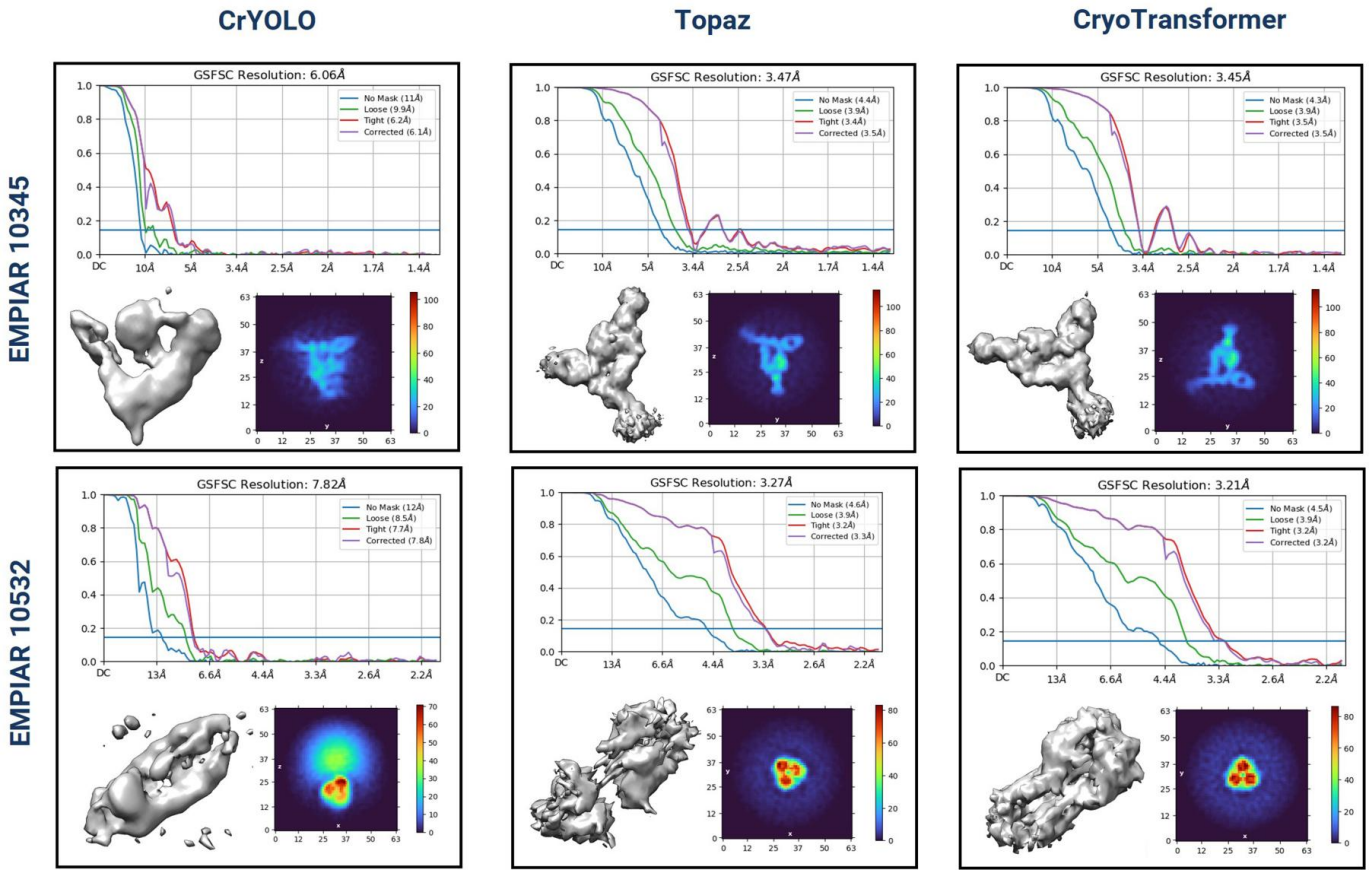
Assessment of CrYOLO, Topaz, and CryoTransformer based on the 3D resolution CSFSC curves, 3D density maps, and density projections on cryo-EM graphs of two proteins (EMPIAR 10345 and 10532). The top diagram in each row shows CSFSC curves, which indicate the resolution of 3D density maps for proteins structures reconstructed from picked particles. Bottom-left image in each sub-figure provides a visual representation of the 3D density map. The bottom-right image in each sub-figure depicts the density projections from the intermediate output of the ab initio reconstruction phase. The integrated density values along the normal direction to that plane are displayed. The color scheme in the heatmap corresponds to the scalar density values at each voxel, with the color intensity indicating density magnitude.

With *Select 2D*, CryoTransformer has the highest resolution of the reconstructed density maps for three out of four proteins (i.e. EMPIAR IDs: 10081, 10532, and 10345), while Topaz has the highest resolution for one protein. Without *Select 2D*, CryoTransformer and Topaz each perform best on two proteins. The detailed assessment of crYOLO, Topaz, and CryoTransformer based on the 3D resolution of Gold Standard Fourier Shell Correlation (FSC) curves, 3D density maps, and density projections with *Select 2D* is visualized in Fig. 2.

In Fig. 2, FSC curves are plotted to assess the resolution of the obtained 3D density maps. Different variations of Fourier Shell Correlation (FSC) plots are presented: one employing an automatically generated mask with a 15 Å falloff, termed the ‘loose mask’ curve, and the other using an auto-generated mask with a falloff of 6 Å for all FSC plots, referred to as the ‘tight mask’ curve. The 3D density map reconstructed by each method for each protein is also visualized. The notable difference between the results of CrYOLO and CryoTransformer can be observed. For instance, in the case of EMPIAR 10345, the correct shape of the density map has three distinct legs (Campbell *et al.* 2020), but CrYOLO failed to capture all three, yielding a lower resolution of 6.06 Å. In contrast, CryoTransformer captured all of them and achieved a high resolution of 3.45 Å. Similarly, in case of EMPIAR 10532, Topaz missed the central segment of the rod-like protein structure, whereas CryoTransformer successfully reconstructed that portion, attaining the highest resolution (3.21 Å) among all methods.

The plot located in the lower-right corner of each section in Fig. 2 represents the intermediate output of the ab-initio reconstruction phase. These plots depict density projections, but instead of slicing the density along a specific plane, the integrated density values along the normal direction to that plane are displayed. The color scheme in the heatmap corresponds to the scalar density values at each voxel, with color intensity indicating density magnitude. Supplementary Figure S8 includes the comparison of the three methods in terms of the quality of 3D density maps for two more proteins.

In addition to this visual assessment in Fig. 2 and Supplementary Fig. S8, we conducted a comparison based on the visual orientation of the picked particles and the 2D classes of the those particles (Supplementary Fig. S9), showing that CryoTransformer picked particles in multiple orientation states that are important for obtaining high-resolution density maps. This analysis specifically involved analyzing the elevation versus azimuth plots for each test EMPIAR IDs. In the case of EMPIAR 10532 in Supplementary Fig. S9, CrYOLO struggled to select particles representing various orientations, resulting in low-quality 2D particle classes. In contrast, Topaz performed reasonably well in picking particles with a diverse range of orientations, and CryoTransformer excelled in picking a substantial number of particles with a broad angular distribution, as indicated by the red color in the heatmap. The higher intensity of the red color in the upper section of each block in Supplementary Fig. S9 corresponds to the higher number of particles in that particular elevation versus azimuth direction. Similarly, the lower section of each block depicts the averaged 2D orientation classes generated from picked particles. The diverse set of particles picked by CryoTransformer enabled the reconstruction of the density map of the highest resolution for this protein.

To further interpret the generated 3D density maps from three different methods, the local resolution maps for them were constructed and analyzed (see Supplementary Fig. S10). As different regions of a 3D density map have variation in resolution, the local resolution analysis indicates how well-defined or detailed a particular region of the map is. High local resolution means that the structural features in that region are well-resolved and can be interpreted with confidence, while low local resolution suggests less detailed and less reliable information.

To estimate the local resolution map, we used CryoSPARC’s *Local Refine* job. Subsequently, the obtained local resolution map was superimposed onto the original density map using Chimera X. A color scale was then employed to depict the local resolution, with high-resolution regions represented in red and low-resolution regions in purple. For instance, in the case of EMPIAR 10532 (refer to Supplementary Fig. S10), the majority of the protein structure regions derived from CrYOLO-picked particles appears in purple and white, while only the tips of the density map exhibit high resolution. This means that CrOYLO was able to capture only a specific set of orientations of particles in micrographs accurately. In contrast, Topaz and CryoTransformer were able to capture a broader range of particle orientations, resulting in a predominantly red-colored density map.

### 3.2 Comparing CryoTransformer, CrYOLO, and Topaz on a subset of micrographs in CryoPPP dataset for the independent test proteins (∼300 micrographs per protein)

Similarly, as in Section 3.1, we compared CryoTransformer, CrYOLO, and Topaz on the labeled subset of micrographs in CryoPPP for the six proteins in the independent test dataset in terms of the resolution of reconstructed density maps. The density maps were reconstructed using the *Select 2D* job from the picked particles. The 3D resolution is listed in Table 2.

**Table 2.** Comparison of CryoTransformer with CrYOLO and Topaz’s performance in terms of the resolution of 3D density maps reconstructed for six test proteins from the particles picked from a small set of micrographs in the CryoPPP.

EMPIAR ID	Number of micrographs	Number of particles			3D resolution (Å)		
		CrYOLO	Topaz	CryoTransformer	CrYOLO	Topaz	CryoTransformer
10017 (Scheres 2015)	84	283	98 625	43 735	10.4	**5.3**	5.61
10081 (Lee and MacKinnon 2017)	300	17 550	111 752	88 632	9.78	7.81	**5.47**
10093 (Jin *et al.* 2017)	295	8802	257 490	151 545	8.8	**6.06**	6.86
10345 (Campbell *et al.* 2020)	295	4095	93 699	105 739	10.2	8.12	**6.43**
10532 (Tan and Rubinstein 2020)	300	12 166	356 222	148 345	15.69	5.47	**3.92**
11056 (Asami *et al.* 2022)	305	46 582	144 600	98 193	10	8.34	**7.42**

Bold font denotes the best resolution of the density map reconstructed from picked particles in the three trials.

Among the six datasets considered, CryoTransformer outperforms crYOLO and Topaz in four instances, despite picking a much smaller number of particles than Topaz in most cases. This observation underscores Topaz's tendency to pick more duplicate particles or false positives. CrYOLO performs substantially worse than CryoTransformer and Topaz because it picks a much small number of particles, which are not sufficient to build good density maps. For the same four proteins, the best resolution of the density maps in Table 2 is lower than that in Table 1 because a much small number of micrographs were used for the particle picking and density map reconstruction.

Our in-depth analysis has revealed the significant impact of increasing the number of particles with wide conformations on the resolution of reconstructed 3D density maps. Notably, augmenting the quantity of micrographs leads to an increased picking of protein particles with diverse conformations, resulting in superior resolution. Conversely, when the number of distinct conformational particles remains constant, increasing the number of micrographs yields no substantial enhancement in the final 3D resolution.

In addition to the evaluation based on 3D resolution and the number of picked particles, we also we compared the particles picked by each method with the ground truth particles labeled in CryoPPP in terms of four machine learning metrics: precision, recall, F1 score, and Dice score. Precision, a measure of prediction accuracy, tells us how well the model avoids false positives. Recall, a measure of the ability to identify relevant instances, assesses the model's avoidance of false negatives. The F1 score, a harmonic mean of precision and recall, strikes a balance between these two metrics. Additionally, Dice score evaluates the overlap between the predicted and true protein particles. The details are listed in Table 3.

**Table 3.** Comparison of CryoTransformer with crYOLO and Topaz in terms of precision, recall, F1-score, and dice score of particle picking on the micrographs of six independent test proteins.

EMPIAR ID	Type of protein	Number of micrographs	No. of particles in ground truth (CryoPPP dataset)	Precision			Recall			F1 score			Dice score		
				CrYOLO	Topaz	CryoTransformer	CrYOLO	Topaz	CryoTransformer	CrYOLO	Topaz	CryoTransformer	CrYOLO	Topaz	CryoTransformer
10017	β-galactosidase	84	49 391	0.695	0.57	**0.745**	0.024	**0.998**	0.587	0.046	**0.726**	0.657	0.041	**0.694**	0.623
10081	Transport	300	39 352	0.405	0.736	**0.860**	0.18	**0.965**	0.889	0.25	0.835	**0.874**	0.214	**0.825**	0.823
10093	Membrane	295	56 394	0.574	**0.617**	0.560	0.054	**0.537**	0.689	0.098	0.574	**0.618**	0.086	0.504	**0.600**
10345	Signaling	295	15 894	0.543	0.526	**0.744**	0.134	**0.981**	0.864	0.215	0.685	**0.799**	0.111	0.659	**0.684**
10532	Viral	300	87 933	0.715	0.672	**0.813**	0.201	**0.976**	0.665	0.313	**0.796**	0.732	0.239	**0.757**	0.614
11056	Transport	305	125 908	0.513	0.731	**0.853**	0.214	**0.832**	0.683	0.302	**0.778**	0.758	0.284	**0.692**	0.679
	Average			0.574	0.642	**0.7625**	0.1345	**0.8815**	0.7295	0.204	0.732	**0.740**	0.163	**0.689**	0.671

CryoTransformer stands out with the highest average precision of 0.7625 and the highest average F1-score of 0.740, indicating that it excels in producing accurate positive predictions and achieves the best-balanced performance considering both precision and recall. Topaz has the highest average recall and dice score of 0.8815 and 0.671, highlighting its ability to correctly identify a high proportion of true positive particles and generate a strong overlap between predicted and actual positive instances.

### 3.3 Visual inspection of particles picked by CryoTransformer, CrYOLO, and Topaz

Supplementary Figure S11 visualizes the particles picked by the three methods from the four representative micrographs of four proteins in the internal test data, which consist of 10% of micrographs from the 80%-10%-10% train-valid-test split (see detailed results in Supplementary Table S10). Consistent with the results in Section 3.2, CrYOLO tends to select few true protein particles, consequently missing many true positive across various protein types. In contrast, Topaz selects an excessive number of particles, often leading to overlaps. Additionally, Topaz frequently picks false particles from contaminations, particle aggregates and ice patches, which can result in lower-resolution 3D density map reconstruction. Furthermore, picking a lot of redundant particles requires much more storage to store them and a lot of time and memory to reconstruct density maps from them. CryoTransformer, on the other hand, often picks most of true particles while keeping false positives to a low level, which is beneficial for 3D density map reconstruction.

## 4 Conclusion

We present CryoTransformer, a novel deep learning method for protein particle recognition and extraction. It leverages the power of transformers, residual networks, traditional image processing techniques, and a bipartite matching loss function. CryoTransformer was trained and tested on the largest labeled particle dataset available. Several ablation studies were conducted to assess the influence of various technical components, including the effect of denoising micrographs. We used diverse metrics such as machine learning metrics, averaged 2D particle resolution, 3D density map resolution, and diversity of particle orientations to comprehensively evaluate the method’s performance. According to the rigorous evaluations and comparisons, CryoTransformer achieves the state-of-the-art performance, making it a robust AI-based tool to automate the process of picking protein particles from cryo-EM micrographs.

## Supplementary Material

btae109_Supplementary_Data

## Data Availability

The source code and data are available at the GitHub repository: https://github.com/jianlin-cheng/CryoTransformer.
