# Supplementary material for: CryoTransformer: a transformer model for picking protein particles from cryo-EM micrographs
Source: Bioinformatics. 2024 Feb 24;40(3):btae109. doi: 10.1093/bioinformatics/btae109 (PMC10937899; doi:10.1093/bioinformatics/btae109)
Supplement: btae109_Supplementary_Data [file btae109_supplementary_data.docx]

**Supplementary Information for**

**CryoTransformer: A Transformer Model for Picking Protein Particles from Cryo-EM Micrographs**

Ashwin Dhakal ^1,2^, Rajan Gyawali ^1,2^, Liguo Wang ^3^, Jianlin Cheng ^1,2,*^

^1^ Department of Electrical Engineering and Computer Science, University of Missouri, Columbia, MO 65211, USA

^2^ NextGen Precision Health, University of Missouri, Columbia, Columbia, MO 65211, USA

^3^ Laboratory for BioMolecular Structure (LBMS), Brookhaven National Laboratory, Upton, NY 11973, USA

*Corresponding author: Jianlin Cheng (chengji@missouri.edu)

**This supplement document contains Supplementary Figures S1-S11, Supplementary Tables S1-S10, and Supplementary Note S1.**

**Supplementary Figures**


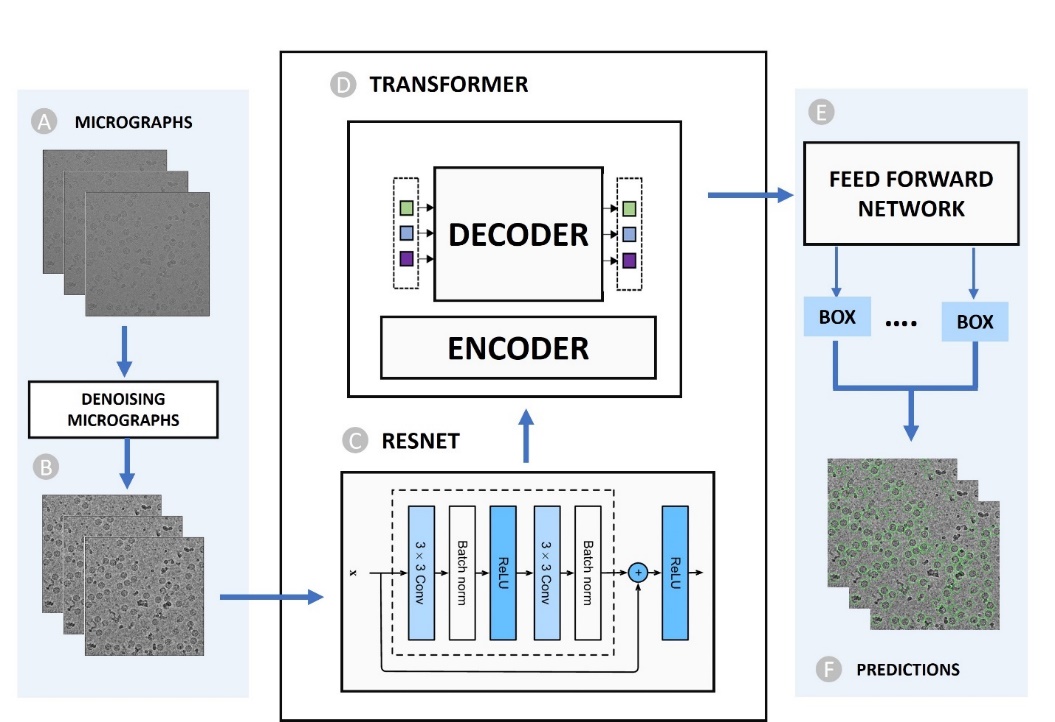


**Supplementary Figure S1**: Overview of the CryoTransformer Particle Picking Pipeline. (A) Input raw micrograph undergoes initial denoising. (B) Denoised micrographs serve as input for subsequent processing. (C) CNN-Based Resnet-152 architecture extracts image features. Features extracted in (C) are processed by an (D) encoder-decoder Transformer. (E) Feed-forward networks further refine the predicted data. (F) Predictions of particles encircled in micrographs, are eventually stored in star files as the final output.


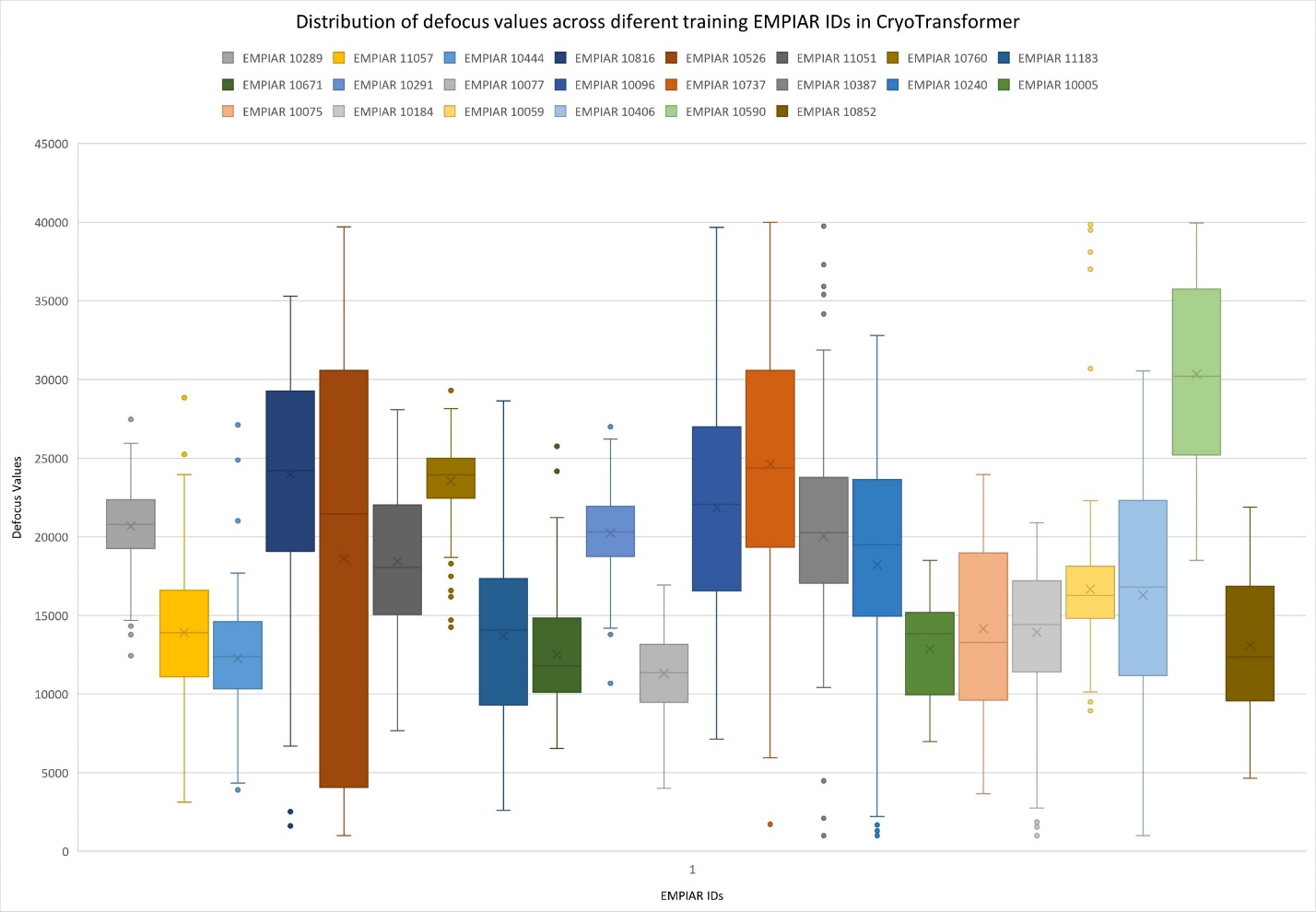


***Supplementary Figure S2:*** *Box and whisker plot showing the distribution of Defocus Values of the training data used in CryoTransformer across different EMPIAR IDs. The central line denotes the defocus value, while the box signifies the interquartile range (IQR). The whiskers extend to encompass values that are 1.5 times the IQR above and below, encompassing the highest and lowest values. Any data points that fall beyond the whiskers are displayed as outliers.*


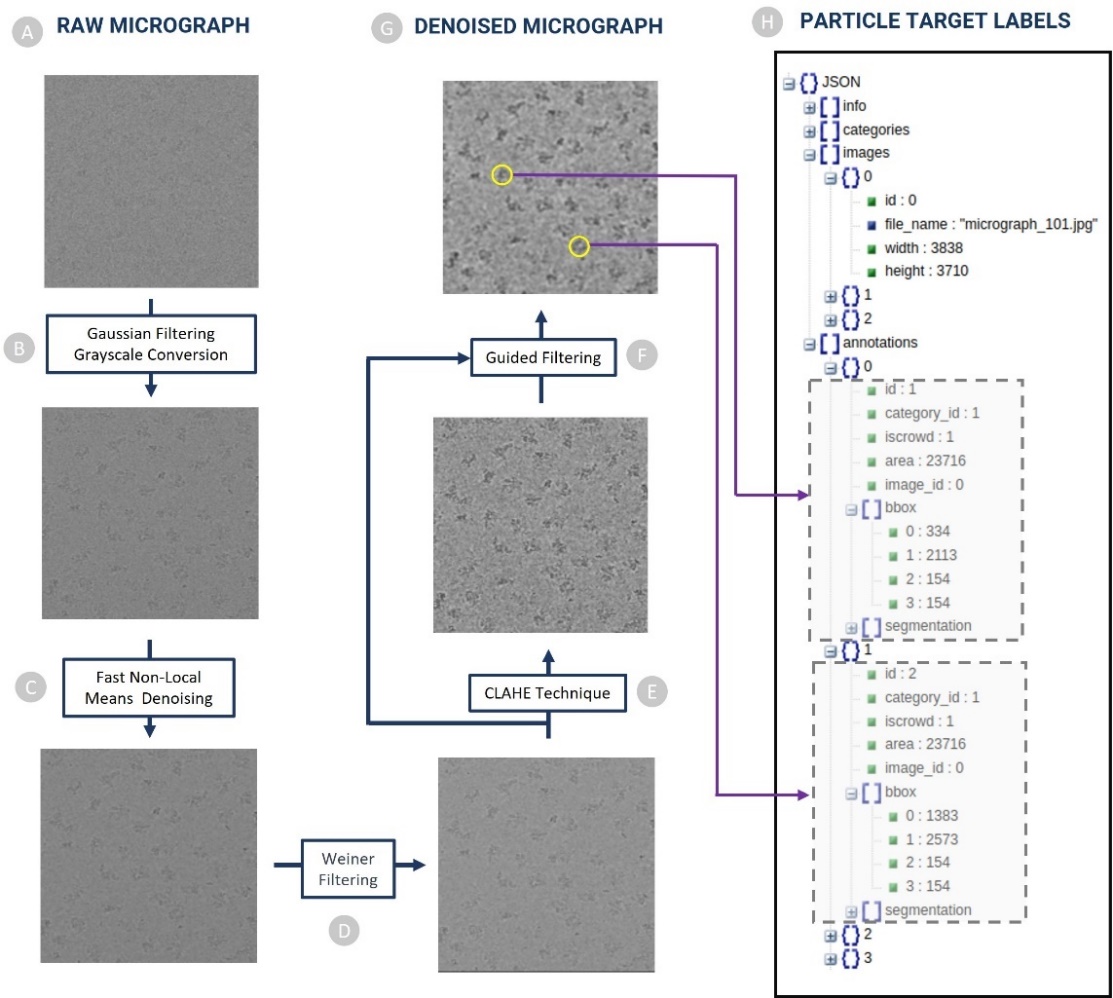


**Supplementary Figure S3**: The denoising used to preprocess cryo-EM micrographs in CryoTransformer. (A) Raw micrographs with low SNR go through (B) Gaussian filtering and Grayscale conversion. Normalized micrographs undergo (C) FastNLMeans denoising technique. (D) Weiner filtering is applied to the micrographs from the previous step, and subsequently (E) CLAHE technique is used to enhance visual clarity of the micrographs. Eventually, (F) Guided filtering is performed using the CLAHE-enhanced micrographs as a guide to obtain (G) denoised micrographs. (H) Ground truth particle annotation data. Particle coordinates from ground truth coordinate files are extracted to create COCO-dataset that is used as target labels for training CryoTransformer.

**Supplementary Figure S4**: Ablation study on the impact of different Backbone Architectures on the CryoTransformer model, trained with Non-Denoised training data (X axis represents different loss functions, Y axis represents amplified loss values ). ResNet 152 performs the best and ResNet 18 performs the worst in terms of all the loss functions used to evaluate the model.

**Supplementary Figure S5**: Ablation study on the effect of varying Backbone Architectures in the CryoTransformer model, trained on Denoised training data (X axis represents different loss functions, Y axis represents amplified loss values). ResNet 152 performs the best and ResNet 18 performs the worst in terms of all the loss functions used to evaluate the model.

**Supplementary Figure S6**: Ablation study on the impact of different datasets (Denoised vs Non-Denoised) on the CryoTransformer model utilizing ResNet 152 as the backbone architecture. Upper: Difference in loss functions through bar plots, Lower: Magnified difference in line graphs. Denoised data outperforms Non-Denoised data across all the loss functions when accessing the model.


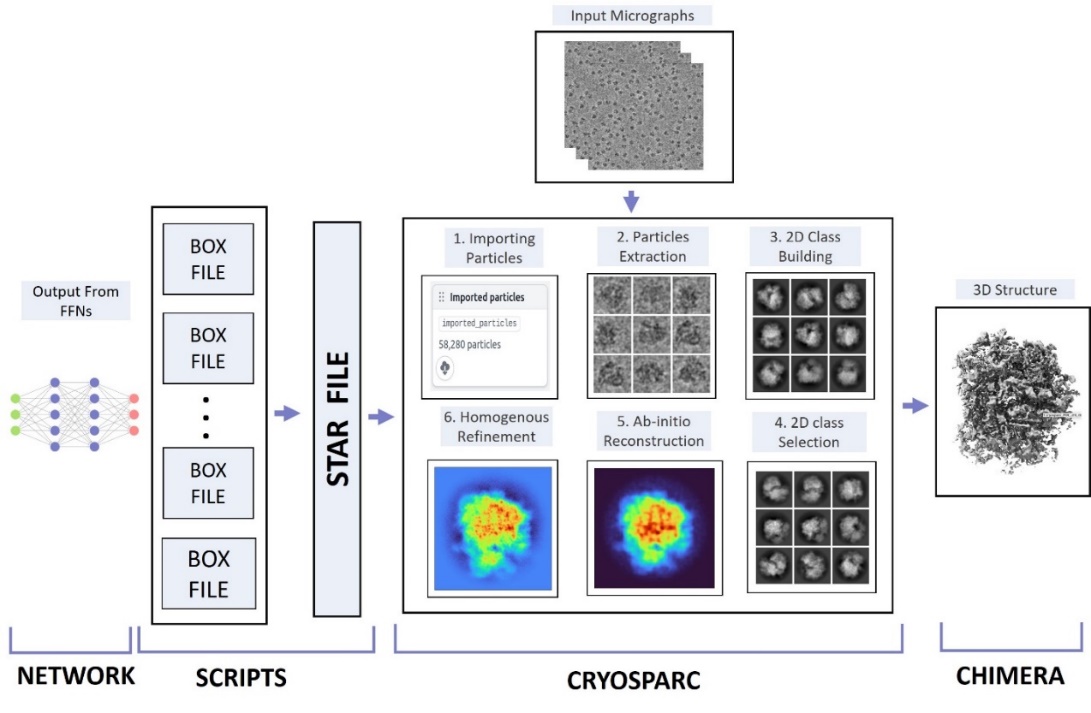


**Supplementary Figure S7**: Post processing steps to generate 3D protein density maps from picked particles. CryoTransformer outputs the .star files, which are imported into CryoSPARC along with the micrographs. Steps (1-6) are performed in CryoSPARC to generate the 3D density maps for a protein. The resolution of the density maps is employed as the main metric to evaluate the quality of the picked particles.


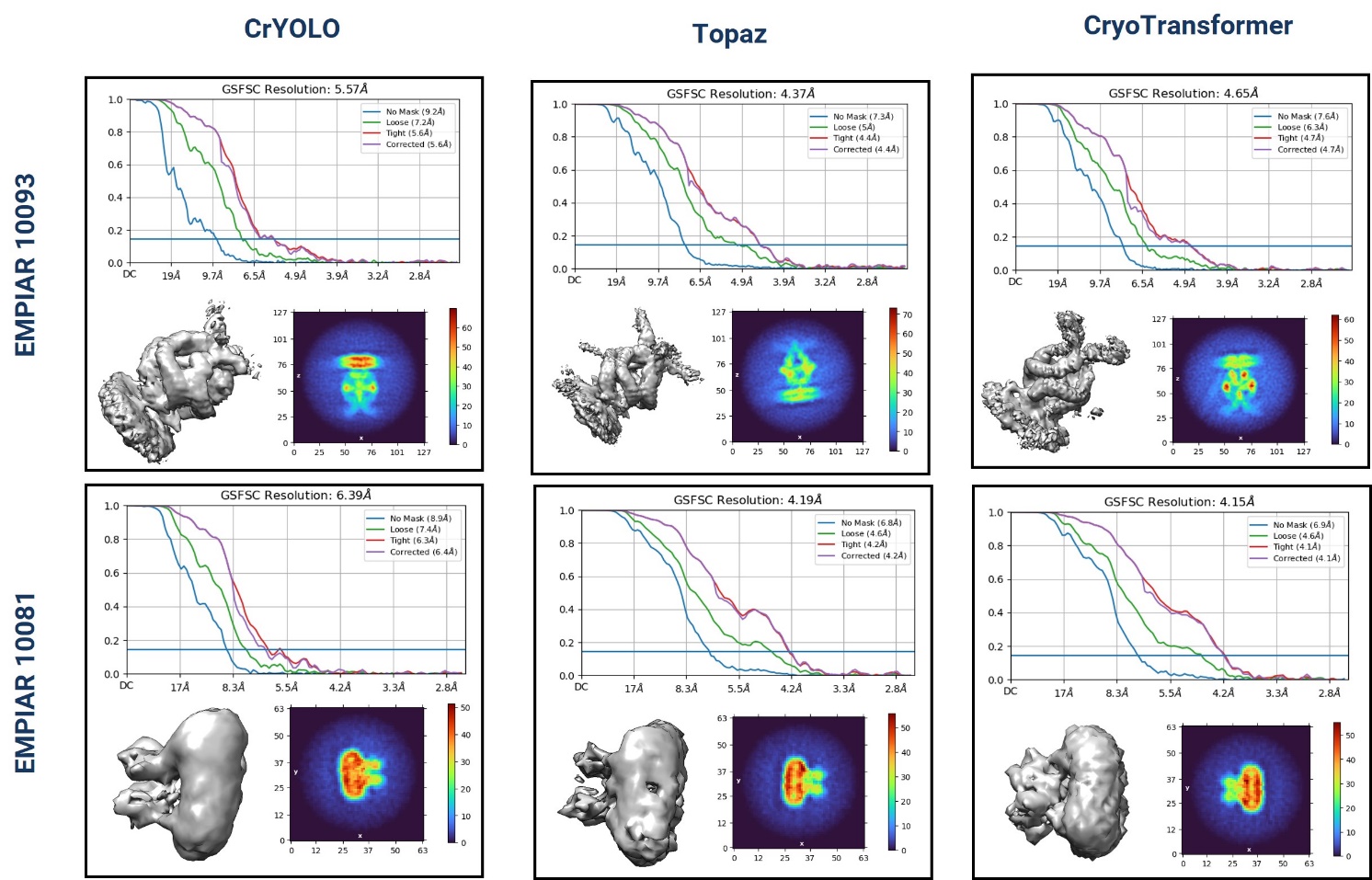


***Supplementary Figure S8:*** *Assessment of CrYOLO, Topaz, and CryoTransformer based on the 3D resolution CSFSC curves, 3D density maps, and density projections for EMPIAR 10093 and EMPIAR 10081.*


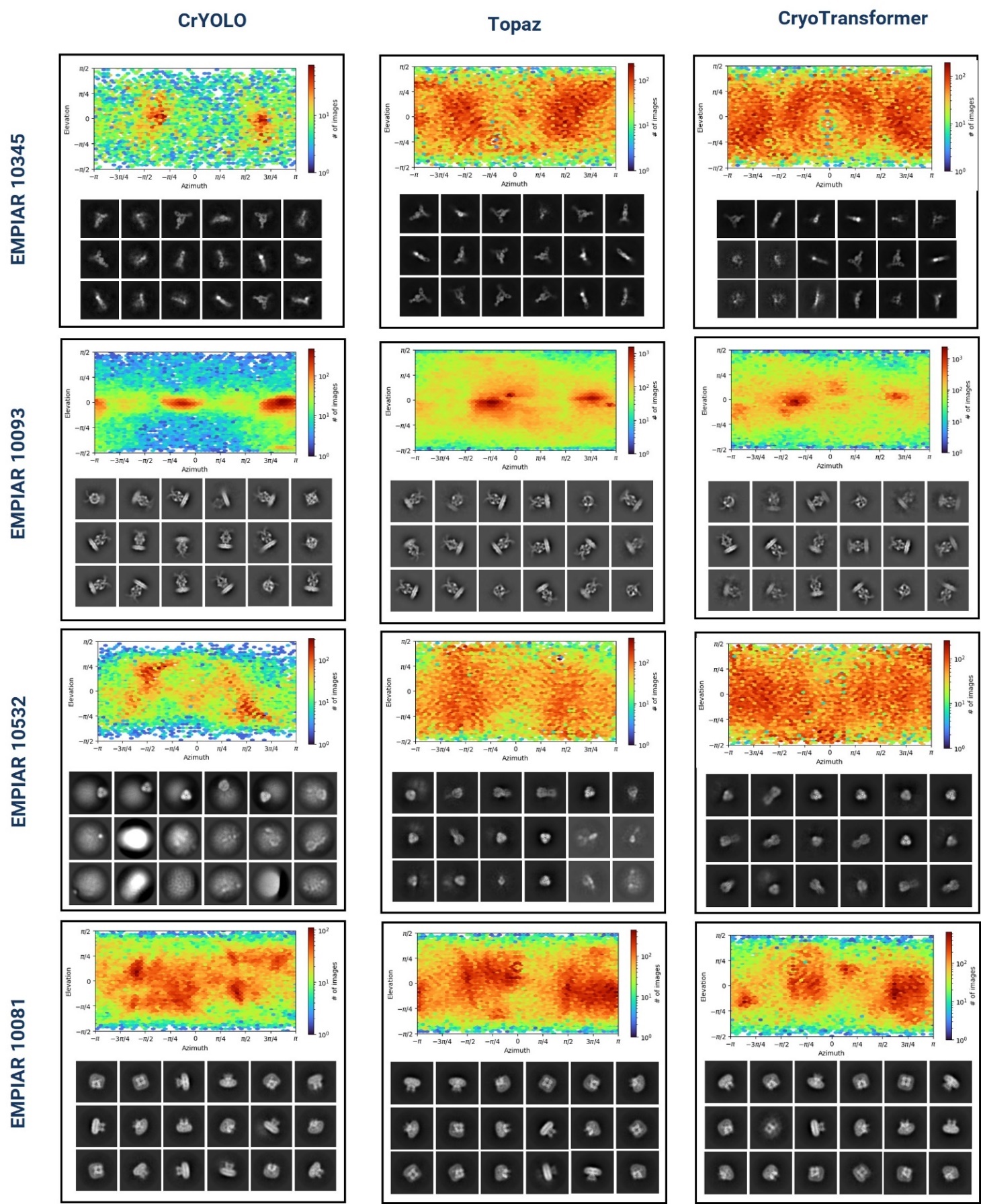


**Supplementary Figure S9**: Assessment of CrYOLO, Topaz, and CryoTransformer based on the visual orientation and 2D classes of the picked protein particles. Each block displays two sections: the upper section presents the viewing direction plots as elevation vs azimuth plots, while the lower section showcases the averaged 2D classes generated from picked particles.


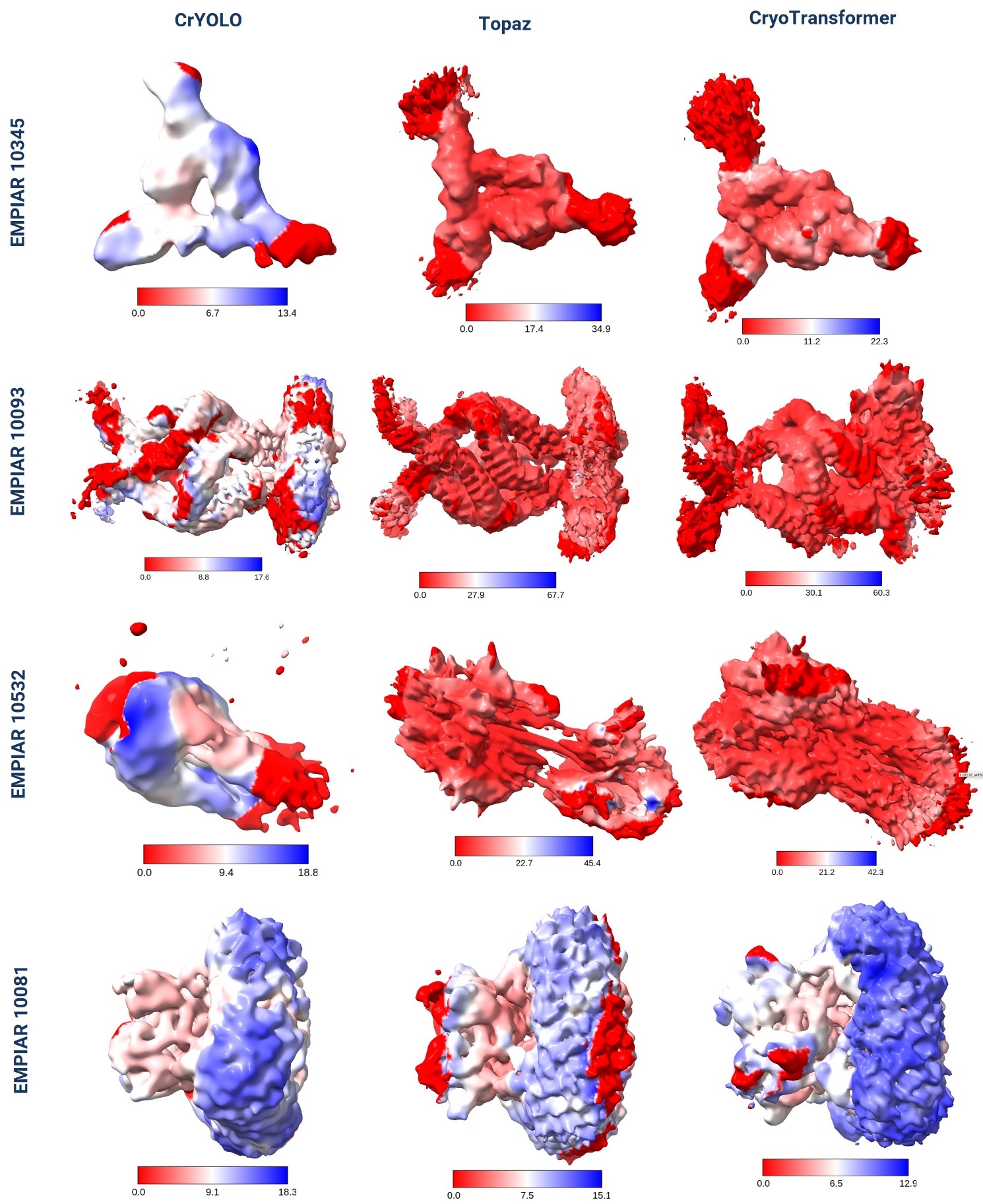


**Supplementary Figure S10**: Assessment of CrYOLO, Topaz, and CryoTransformer based on the local resolution analysis of 3D density maps. The color scale (in Angstrom) employed represents high-resolution areas in red and low-resolution regions in purple.


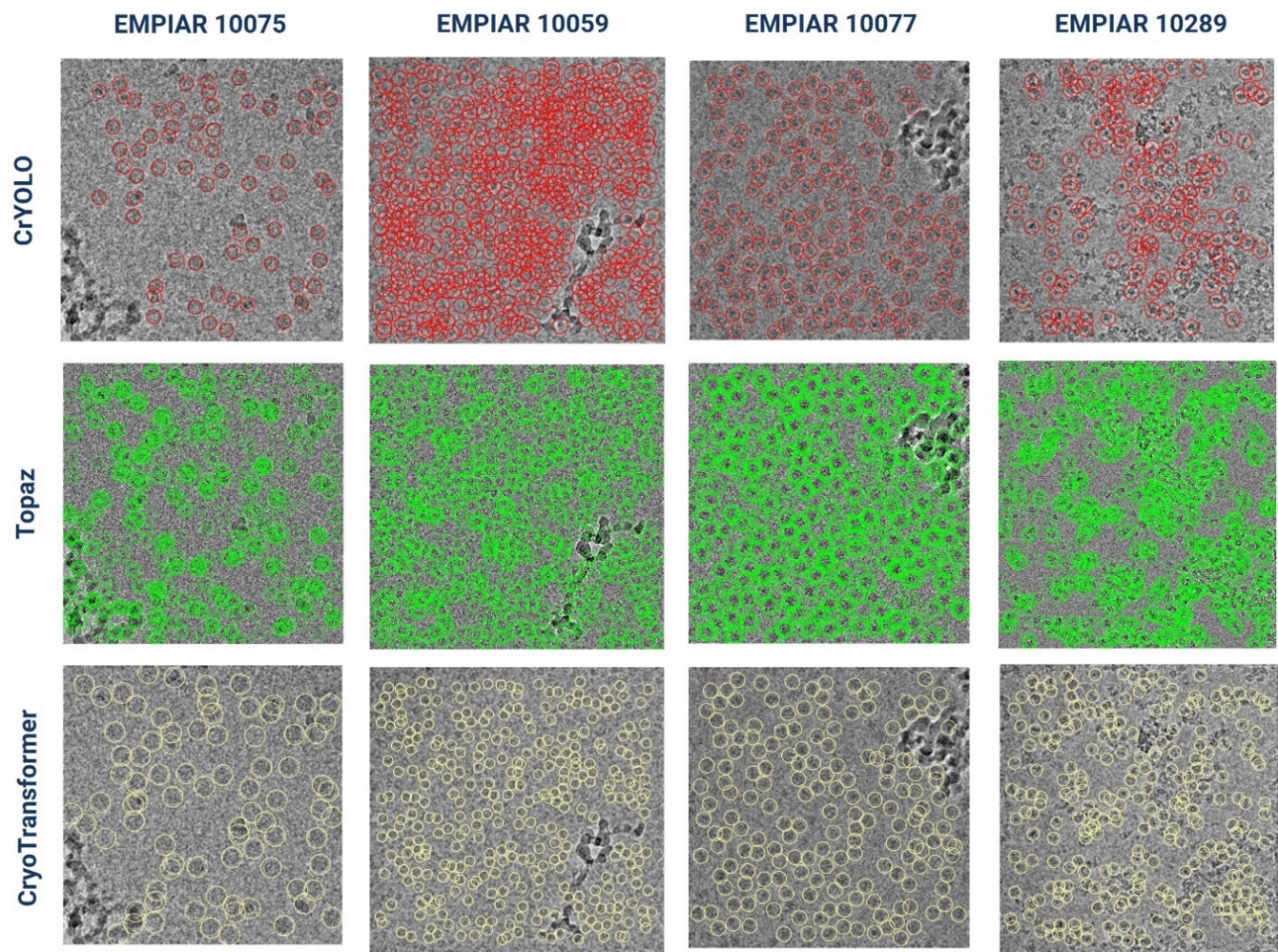


**Supplementary Figure S11**: Assessment of CrYOLO, Topaz, and CryoTransformer based on visual inspection of predicted particles in micrographs of four typical proteins. The first row (indicated by red circles) represents protein particles picked by CrYOLO. The second row (marked by green circles) displays protein particles picked by Topaz. The third row (with yellow circles) illustrates protein particles picked by CryoTransformer.

**Supplementary Tables**

**Supplementary Table S1:** The statistics and information of the 22 sets of micrographs for training, validation, and internal test of CryoTransformer (* Theoretical weight)

| **SN** | **EMPIAR ID** | **Type of Protein** | **Image Size** | **Total Structure Weight (kDa)** | **# Training Micrographs** | **# Validation Micrographs** | **# Test Micrographs** | **# Total Micrographs** |
| --- | --- | --- | --- | --- | --- | --- | --- | --- |
| 1 | 11183​ (Liu *et al.*, 2022) | Signaling Protein | (5760, 4092) | 139.36 | 250 | 25 | 25 | 300 |
| 2 | 11057​ (Tanaka *et al.*, 2022) | Hydrolase | (5760, 4092) | 149.43 | 250 | 25 | 20 | 295 |
| 3 | 11051​ (Newing *et al.*, 2020) | Transcription/DNA/RNA | (3838, 3710) | 357.31 | 250 | 25 | 25 | 300 |
| 4 | 10852​ (Cao *et al.*, 2021) | Signaling Protein | (5760, 4092) | 157.81 | 270 | 40 | 33 | 343 |
| 5 | 10816​ (Oldham *et al.*, 2016) | Transport Protein | (7676, 7420) | 166.62 | 250 | 25 | 25 | 300 |
| 6 | 10760​ (Kuzuya *et al.*, 2022) | Membrane Protein | (3838, 3710) | 321.69 | 250 | 25 | 25 | 300 |
| 7 | 10737​ (Li *et al.*, 2021) | Membrane Protein | (5760, 4092) | 155.83 | 250 | 25 | 17 | 292 |
| 8 | 10671​ (Josephs *et al.*, 2021) | Signaling Protein | (5760, 4092) | 77.14 | 250 | 25 | 23 | 298 |
| 9 | 10590​ (Pettersen *et al.*, 2021) | Transport Protein | (3710, 3838) | 1000* | 250 | 25 | 21 | 296 |
| 10 | 10526​ (Li *et al.*, 2020) | Ribosome (50S) | (7676, 7420) | 1085.81 | 180 | 20 | 20 | 220 |
| 11 | 10444​ (Demura *et al.*, 2020) | Membrane Protein | (5760, 4092) | 295.89 | 250 | 25 | 21 | 296 |
| 12 | 10406​ (Nicholson *et al.*, 2020) | Ribosome (70S) | (3838, 3710) | 632.89 | 200 | 20 | 19 | 239 |
| 13 | 10387​ (Passos *et al.*, 2020) | Viral Protein | (3710, 3838) | 185.87 | 250 | 25 | 24 | 299 |
| 14 | 10291​ (Burendei *et al.*, 2020) | Transport Protein | (3710, 3838) | 361.39 | 250 | 25 | 25 | 300 |
| 15 | 10289​ (Burendei *et al.*, 2020) | Transport Protein | (3710, 3838) | 361.39 | 250 | 25 | 25 | 300 |
| 16 | 10240​ (Falzone *et al.*, 2019) | Lipid Transport Protein | (3838, 3710) | 171.72 | 250 | 25 | 24 | 299 |
| 17 | 10184​ (Kim *et al.*, 2018) | Aldolase | (3838, 3710) | 150* | 250 | 25 | 21 | 296 |
| 18 | 10096​ (Zi Tan *et al.*, 2017) | Viral Protein | (3838, 3710) | 150* | 250 | 25 | 25 | 300 |
| 19 | 10077​ (Fischer *et al.*, 2016) | Ribosome (70S) | (4096, 4096) | 2198.78 | 250 | 25 | 25 | 300 |
| 20 | 10075​ (Koning *et al.*, 2016) | Bacteriophage MS2 | (4096, 4096) | 1000* | 250 | 25 | 24 | 299 |
| 21 | 10059​ (Gao *et al.*, 2016) | Transport Protein | (3838, 3710) | 317.88 | 250 | 25 | 16 | 291 |
| 22 | 10005​ (Liao *et al.*, 2013) | Transport Protein | (3710, 3710) | 272.97 | 22 | 4 | 3 | 29 |
|  |  | **Total Micrographs** |  |  | **5,172** | **534** | **486** | **6,192** |

***Supplementary Table S2:*** *Data statistics used for independent Testing sourced from EMPIAR repository.*

| **SN** | **EMPIAR ID** | **Type of Protein** | **Source Organism** | **Micrograph Size** | **Total Structure Weight (kDa)** | **Number of Micrographs** |
| --- | --- | --- | --- | --- | --- | --- |
| 1 | 10081 | Transport Protein | Homo sapiens | (3710, 3838) | 298.57 | 997 |
| 2 | 10532 | Viral Protein | Influenza A virus subtype H3N2 | (4096, 4096) | 191.76 | 1,556 |
| 3 | 10093 | Membrane Protein | Drosophila melanogaster | (3838, 3710) | 779.4 | 1,873 |
| 4 | 10345 | Signaling Protein | Mus musculus | (3838, 3710) | 244.68 | 1,644 |
|  |  | **Total Micrographs** |  |  |  | 6,070 |

***Supplementary Table S3:*** *Data statistics used for independent Testing sourced from CryoPPP dataset (* Theoretical weight)*

| **SN** | **EMPIAR ID** | **Type of Protein** | **Source Organism** | **Micrograph Size** | **Total Structure Weight (kDa)** | **Number of Micrographs** |
| --- | --- | --- | --- | --- | --- | --- |
| 1 | 10017 | β -galactosidase | Escherichia coli K-12 | (4096, 4096) | 450* | 84 |
| 2 | 10081 | Transport Protein | Homo sapiens | (3710, 3838) | 298.57 | 300 |
| 3 | 10093 | Membrane Protein | Drosophila melanogaster | (3838, 3710) | 779.4 | 295 |
| 4 | 10345 | Signaling Protein | Mus musculus | (3838, 3710) | 244.68 | 295 |
| 5 | 10532 | Viral Protein | Influenza A virus subtype H3N2 | (4096, 4096) | 191.76 | 300 |
| 6 | 11056 | Transport Protein | Mus musculus | (5760, 4092) | 88.94 | 305 |
|  |  | **Total Micrographs** |  |  |  | 1,579 |

**Supplementary Table S4:** CryoTransformer model configuration and tuned hyperparameter values.

| **Parameters** | **Type** | **Value** | **Description** |
| --- | --- | --- | --- |
| lr | float | 0.0001 | Learning rate |
| bbox_loss_coef | float | 5 | Weight assigned to the bounding box regression loss |
| clip_max_norm | float | 0.1 | Gradient clipping max norm |
| dec_layers | int | 6 | Number of decoding layers in the transformer |
| dice_loss_coef | float | 1 | Weight assigned to the Dice loss |
| dilation | Boolean | FALSE | If true, replace stride with dilation in the last convolutional block (DC5) |
| dim_feedforward | int | 2048 | Intermediate size of the feedforward layers in the transformer blocks |
| dropout | float | 0.1 | Dropout applied in the transformer |
| enc_layers | int | 6 | Number of encoding layers in the transformer |
| eos_coef | float | 0.1 | Relative classification weight of the no-object class |
| giou_loss_coef | float | 2 | Weight assigned to the Generalized Intersection over Union (GIOU) loss |
| hidden_dim | int | 256 | Size of the embeddings (dimension of the transformer |
| lr_backbone | float | 0.00001 | Learning rate for the backbone network |
| lr_drop | int | 150 | Learning rate drop factor. |
| mask_loss_coef | float | 1 | Weight assigned to the mask loss |
| nheads | int | 8 | Number of attention heads inside the transformer's attentions |
| num_queries | int | 600 | Number of query slots |
| position_embedding | str | sine | Type of positional embedding to use on top of the image features |
| set_cost_bbox | float | 5 | L1 box coefficient in the matching cost |
| set_cost_class | float | 1 | Class coefficient in the matching cost |
| set_cost_giou | float | 2 | GIoU box coefficient in the matching cost |
| weight_decay | float | 0.0001 | L2 regularization |
| world_size | int | 1 | Number of distributed processes |

**Supplementary Table S5:** Results from the ablation study on the effect of varying Backbone Architectures in the CryoTransformer model trained on Non-Denoised training data. ResNet 152 performs the best in terms of all the loss functions used to evaluate the model.

| **Backbone Architecture** | **Train Loss (Overall)** | **Train Loss (Bounding Box)** | **Train Loss (GIoU)** | **Val Loss (Overall)** | **Val Loss (Bounding Box)** | **Val Loss (GIoU)** | **# Epochs** | **# Parameters** | **Learning Rate** |
| --- | --- | --- | --- | --- | --- | --- | --- | --- | --- |
| ResNet18 | 18.190 | 0.591 | 4.499 | 34.097 | 3.125 | 4.537 | 50 | 28791558 | 0.00001 |
| ResNet34 | 18.168 | 0.489 | 4.496 | 32.077 | 3.116 | 3.538 | 50 | 38818566 | 0.00001 |
| ResNet50 | 14.194 | 0.294 | 3.497 | 30.514 | 2.129 | 3.519 | 50 | 41407238 | 0.00001 |
| ResNet101 | 13.375 | 0.209 | 2.509 | 27.310 | 2.103 | 2.522 | 50 | 60347142 | 0.00001 |
| ResNet152 | 11.768 | 0.189 | 1.514 | 24.123 | 1.781 | 2.576 | 50 | 75944710 | 0.00001 |

**Supplementary Table S6:** Statistics of ablation study on the impact of varying Backbone Architectures in the CryoTransformer model trained on Denoised training data. ResNet 152 performs the best in terms of all the loss functions used to evaluate the model.

| **Backbone Architecture** | **Train Loss (Overall)** | **Train Loss (Bounding Box)** | **Train Loss (GIoU)** | **Val Loss (Overall)** | **Val Loss (Bounding Box)** | **Val Loss (GIoU)** | **# Epochs** | **# Parameters** | **Learning Rate** |
| --- | --- | --- | --- | --- | --- | --- | --- | --- | --- |
| resnet18 | 17.690 | 0.482 | 3.423 | 32.030 | 2.902 | 3.300 | 50 | 28791558 | 0.00001 |
| resnet34 | 16.746 | 0.488 | 3.427 | 31.782 | 2.866 | 3.247 | 50 | 38818566 | 0.00001 |
| resnet50 | 13.749 | 0.386 | 2.429 | 32.274 | 2.071 | 2.310 | 50 | 41407238 | 0.00001 |
| resnet101 | 12.785 | 0.190 | 1.433 | 26.341 | 1.968 | 2.342 | 50 | 60347142 | 0.00001 |
| resnet152 | 10.699 | 0.183 | 1.042 | 19.072 | 0.878 | 1.280 | 50 | 75944710 | 0.00001 |

**Supplementary Table S7:** Statistics of ablation study on the effect of varying dataset (Denoised vs Non-Denoised) in the CryoTransformer model with ResNet 152 as backbone architecture. Denoised data performs the best in terms of all the loss functions used to evaluate the model.

| **Input Micrographs** | **Train Loss (Overall)** | **Train Loss (Bounding Box)** | **Train Loss (GIoU)** | **Val Loss (Overall)** | **Val Loss (Bounding Box)** | **Val Loss (GIoU)** | **# Epochs** | **# Parameters** |
| --- | --- | --- | --- | --- | --- | --- | --- | --- |
| Non Denoised | 7.055 | 0.066 | 0.789 | 9.625 | 0.095 | 1.021 | 300 | 75944710 |
| Denoised | 3.952 | 0.062 | 0.752 | 5.418 | 0.093 | 1.004 | 300 | 75944710 |

**Supplementary Table S8:** Topaz parameters and their values used while evaluation.

| **Parameter** | **Value** | **Description** |
| --- | --- | --- |
| Pretrained Model | Resnet 16 | Pretrained model used for extraction |
| Downsampling factor | 4 | Rescaling factor to downsample images by |
| Number of iterations | 200 | Number of iterations to use for model fit |
| Score threshold | 0 | Threshold particles by score before preprocessing |
| Number of parallel processes | 8 | Number of Topaz processes to distribute preprocessing over |
| Radius of extracted regions | 7 | Radius of regions to extract from micrograph |
| Extraction threshold | -6 | Log likelihood score at which to end extraction |
| Particle threshold | 0 | Threshold at which to filter particles |
| Max assignment radius | -1 | Maximum allowed radius for matching prediction to labeled target. |
| Min extraction radius | 5 | Minimum radius to extract from |
| Max extraction radius | 100 | Maximum radius to extract from |
| Step radius | 5 | Grid size when searching for optimal radius |

***Supplementary Table S9:*** *Detailed results of three trials for Comparison of CryoTransformer against CrYOLO and Topaz’s performance in terms of 3D resolution and picked particles count using micrographs from EMPIAR repository.*

| **EMPIAR ID** | **Number of Micrographs** | **CrYOLO** | | | | | | | | **Topaz** | | | | | | | | **CryoTransformer** | | | | | | | |
| --- | --- | --- | --- | --- | --- | --- | --- | --- | --- | --- | --- | --- | --- | --- | --- | --- | --- | --- | --- | --- | --- | --- | --- | --- | --- |
|  |  | **Without Select 2D** | | | | **With Select 2D** | | | | **Without Select 2D** | | | | **With Select 2D** | | | | **Without Select 2D** | | | | **With Select 2D** | | | |
|  |  | **Resolution for 3 Trials (A)** | | | **Number of Particles** | **Resolution for 3 Trials (A)** | | | **Number of Particles** | **Resolution for 3 Trials (A)** | | | **Number of Particles** | **Resolution for 3 Trials (A)** | | | **Number of Particles** | **Resolution for 3 Trials (A)** | | | **Number of Particles** | **Resolution for 3 Trials (A)** | | | **Number of Particles** |
|  |  | **1** | **2** | **3** |  | **1** | **2** | **3** |  | **1** | **2** | **3** |  | **1** | **2** | **3** |  | **1** | **2** | **3** |  | **1** | **2** | **3** |  |
| 10081 | 997 | **7.45** | 7.59 | 7.56 | 59,559 | **6.39** | 6.51 | 6.75 | 32,472 | **6.34** | 6.39 | 6.06 | 383,558 | 4.21 | 4.25 | **4.19** | 148,378 | 5.66 | 5.37 | **4.89** | 293,980 | 4.21 | 4.17 | **4.15** | 147,662 |
| 10532 | 1,556 | 8.59 | 8.58 | **8.34** | 62,732 | **7.82** | 7.93 | 7.9 | 16,079 | **3.97** | 4.41 | 4.12 | 1,574,179 | 3.37 | **3.27** | 3.41 | 260,266 | **3.86** | 3.87 | 3.89 | 764,215 | **3.21** | 3.23 | 3.35 | 259,757 |
| 10093 | 1,873 | 6.26 | 6.28 | **6** | 53,482 | **5.57** | 5.82 | 5.87 | 40,374 | 4.75 | **4.72** | 5.03 | 791,064 | **4.37** | 4.44 | 4.71 | 359,619 | 6.22 | 6.2 | **6.11** | 596,192 | 5.03 | 5.01 | **4.65** | 204,355 |
| 10345 | 1,644 | 7.59 | **7.27** | 7.61 | 19,836 | **6.06** | 6.59 | 7.92 | 5,377 | **3.5** | 3.5 | 3.51 | 396,882 | 3.92 | 3.52 | **3.47** | 155,023 | **5.22** | 5.39 | 5.25 | 182,397 | 3.51 | **3.45** | 3.48 | 111,375 |

***Supplementary Table S10:*** *Comparison of CryoTransformer with crYOLO and Topaz’s performance in terms of 3D resolution and picked particles count using test micrographs from train-valid-test split.*

| **EMPIAR ID** | **Number of Micrographs** | **Number of Protein Particles** | | | **3D Resolution (Å)** | | |  |
| --- | --- | --- | --- | --- | --- | --- | --- | --- |
|  |  |  |  |  |  |  |  |  |
|  |  | **CrYOLO** | **Topaz** | **CryoTransformer** | **CrYOLO** | **Topaz** | **CryoTransformer** |  |
| 10852 | 33 | 64,737 | 27,891 | 19,479 | 7.13 | 8.93 | 5.47 |  |
| 10184 | 21 | 26,947 | 25,743 | 12,167 | 6.49 | 6.83 | 6.32 |  |
| 10240 | 24 | 1,993 | 11,979 | 10,245 | 18.35 | 9.72 | 9.69 |  |
| 10816 | 25 | 1,700 | 1898 | 10,973 | 19.57 | 16.91 | 14.12 |  |
| 10671 | 23 | 45,456 | 6,775 | 13,346 | 7.93 | 5.02 | 4.92 |  |
| 10005 | 3 | 706 | 1,411 | 1,516 | 6.48 | 6.38 | 6.46 |  |
| 10291 | 25 | 13,505 | 36,288 | 13,038 | 8.7 | 9.6 | 8.5 |  |
| 10387 | 24 | 655 | 37,859 | 13,028 | 22.29 | 9.29 | 7.9 |  |
| 10406 | 19 | 2,599 | 23,784 | 3,675 | 4.94 | 4.55 | 4.29 |  |
| 11183 | 25 | 19,001 | 28,195 | 13,689 | 14.55 | 7.53 | 9.33 |  |
| 10737 | 17 | 2,599 | 12,025 | 7,236 | 6.21 | 10.35 | 5.84 |  |

**Supplementary Notes**

***Supp Note S1:*** *Denoising Cryo-EM Micrographs*

The cryo-EM micrographs in *.mrc* format, serve as the initial input for CryoTransformer (**Supplementary** **Figure S2A**). To reduce noise and improve the SNR, a Gaussian filter with a kernel size of 9 is applied to convolve with the images. Subsequently, the images undergo standard normalization to achieve consistent intensity ranges. The normalized pixel values of the images are computed using the formula [pixel = (pixel- μ)/ σ], ensuring that the data is centered and scaled appropriately for further analysis. The normalized images are then converted to grayscale, which collapses multi-channel intensity information into a single channel, ensuring a uniform representation of pixel values ranging from 0 to 255 (**Supplementary** **Figure S2B**).

Effective noise reduction is essential to reveal clear structural details in cryo-EM micrographs. We employ a two-step denoising process to the normalized images, involving Fast Non-Local Means (FastNLMeans) denoising (**Supplementary** **Figure S2C**) followed by Weiner filtering. FastNLMeans denoising is employed to retain image details while suppressing noise artifacts. By exploiting the redundancy present in natural images, FastNLMeans replaces the noisy pixel with a weighted average of similar pixels from a larger neighborhood. The trade-off between noise suppression and detail preservation is controlled by the choice of template window size (7 in this case) and the search window size (21 in this case).

The output of FastNLMeans denoising is subjected to Weiner filtering to further reduce the residual noise and enhance the image's structural fidelity (**Supplementary** **Figure S2D**). It achieves this by estimating the original image's frequency spectrum and applying a correction factor to mitigate the effects of noise. Enhancing contrast in cryo-EM micrographs is crucial for improving particle visibility and overall image quality. We incorporate the Contrast Limited Adaptive Histogram Equalization (CLAHE) technique for this purpose (**Supplementary** **Figure S2E**). The CLAHE technique, with a clip limit of 2 and a tile grid size of 16x16, is applied to the denoised images. This technique effectively addresses non-uniform illumination and low contrast, leading to enhanced visual clarity.

To accomplish selective smoothing and fine detail preservation, guided filtering is performed using the CLAHE-enhanced image as a guide (**Supplementary** **Figure S2F**). Guided filtering operates by estimating the local linear relationship between the guidance image and the target image. This relationship is then used to determine the filtering weights applied to each pixel, resulting in controlled smoothing, while retaining sharp edges and fine details. The filtering fine-tunes the micrographs (**Supplementary** **Figure S2G**), achieving a balance between noise reduction and preservation of important structural information.

References

Burendei,B. *et al.* (2020) Cryo-EM structures of undocked innexin-6 hemichannels in phospholipids. *Sci. Adv.*, **6**.

Cao,C. *et al.* (2021) Structure, function and pharmacology of human itch GPCRs. *Nature*, **600**, 170–175.

Demura,K. *et al.* (2020) Cryo-EM structures of calcium homeostasis modulator channels in diverse oligomeric assemblies. *Sci. Adv.*, **6**, 1–12.

Falzone,M.E. *et al.* (2019) Structural basis of Ca2+-dependent activation and lipid transport by a TMEM16 scramblase. *Elife*, **8**, 1–25.

Fischer,N. *et al.* (2016) The pathway to GTPase activation of elongation factor SelB on the ribosome. *Nature*, **540**, 80–85.

Gao,Y. *et al.* (2016) TRPV1 structures in nanodiscs reveal mechanisms of ligand and lipid action. *Nature*, **534**, 347–351.

Josephs,T.M. *et al.* (2021) Structure and dynamics of the CGRP receptor in apo and peptide-bound forms. *Science (80-. ).*, **372**.

Kim,L.Y. *et al.* (2018) Benchmarking cryo-EM single particle analysis workflow. *Front. Mol. Biosci.*, **5**.

Koning,R.I. *et al.* (2016) Asymmetric cryo-EM reconstruction of phage MS2 reveals genome structure in situ. *Nat. Commun.*, **7**, 1–6.

Kuzuya,M. *et al.* (2022) Structures of human pannexin-1 in nanodiscs reveal gating mediated by dynamic movement of the N terminus and phospholipids. *Sci. Signal.*, **15**, 1–11.

Li,J. *et al.* (2021) Cryo-EM structures of Escherichia coli cytochrome bo3 reveal bound phospholipids and ubiquinone-8 in a dynamic substrate binding site. *Proc. Natl. Acad. Sci. U. S. A.*, **118**.

Li,Q. *et al.* (2020) Synthetic group A streptogramin antibiotics that overcome Vat resistance. *Nature*, **586**, 145–150.

Liao,M. *et al.* (2013) Structure of the TRPV1 ion channel determined by electron cryo-microscopy. *Nature*, **504**, 107–112.

Liu,Y. *et al.* (2022) Ligand recognition and allosteric modulation of the human MRGPRX1 receptor. *Nat. Chem. Biol.*, **19**.

Newing,T.P. *et al.* (2020) Molecular basis for RNA polymerase-dependent transcription complex recycling by the helicase-like motor protein HelD. *Nat. Commun.*, **11**, 1–11.

Nicholson,D. *et al.* (2020) Structure of the 70S Ribosome from the Human Pathogen Acinetobacter baumannii in Complex with Clinically Relevant Antibiotics. *Structure*, **28**, 1087-1100.e3.

Oldham,M.L. *et al.* (2016) Structure of the transporter associated with antigen processing trapped by herpes simplex virus. *Elife*, **5**, 1–16.

Passos,D.O. *et al.* (2020) Structural basis for strand-transfer inhibitor binding to HIV intasomes. *Science (80-. ).*, **367**, 810–814.

Pettersen,E.F. *et al.* (2021) UCSF ChimeraX: Structure visualization for researchers, educators, and developers. *Protein Sci.*, **30**, 70–82.

Tanaka,S. *et al.* (2022) Structural Basis for Binding of Potassium-Competitive Acid Blockers to the Gastric Proton Pump. *J. Med. Chem.*, **65**, 7843–7853.

Zi Tan,Y. *et al.* (2017) Addressing preferred specimen orientation in single-particle cryo-EMthrough tilting. *Nat. Methods*, **14**, 793–796.
